# Mining in Hot Places

**Published:** 1882-11

**Authors:** 


### MINING IN HOT PLACES.

If there are to be found anywhere in the world a set of human salamanders, we may claim the credit of having them here on the Comstock. What would scorch a man who lives wholly on the surface chills a miner inured to the heat of the lower levels. A miner who has been for some months past working in one of the hottest sections of the Comstock, a day or two since gave a reporter his experience of the heat which miners are often called upon to encounter. He says that in working at points where the thermometer marks a temperature of 115 to 120 degrees, great thirst is experienced. No ice water is too cold to be swallowed with a relish. Men go to the water barrel in which huge chunks of ice are floating about, and with their picks chop up the ice in order that the water may be rendered colder by being filled with fine fragments. Often this does not satisfy them, and they chew and swallow lumps of ice.

The natural temperature of the human body—of the blood—is about ninety-eight degrees, Fahrenheit; therefore, when a man remains in a hot place for an hour, or even half an hour, his blood, and his whole body, becomes heated to a temperature of 115, 120 degrees, or whatever may be the temperature of the place in which he is at work. It is then that the miner begins to pour down ice water and eat ice. The strangest thing about the business is that it does not hurt any of the men. Often they swallow such quantities of ice water that their stomachs will not retain it. All they do on such occasions is to swallow more of the same water, but more cautiously.

When the men are working in extremely hot places the temperature of the place to which they come to cool off—the cooling-off station—is probably 100 degrees. This temperature—which would almost roast a surface man—appears cool to a man who has come from a place where the thermometer marks 110 to 115 degrees. In a place where the temperature is ninety the man will feel so cold as to shiver. Often at the cooling-off station, where the temperature is 100 degrees, the perspiration will cease and the man will begin to feel very uncomfortable. On leaving and going back to where the temperature is from 115 to 120, as the perspiration begins to start, there is for a minute or two an intolerable itching over the whole body. As each closed pore re-opens it produces a tingling sensation. However, as soon as there is a free flow of perspiration all this trouble ceases, and the man feels quite comfortable and so remains until his whole body—blood, flesh and bones—again reaches a temperature of from 110 to 120, when he becomes wild for ice water and ice. It is not a little strange—when we consider that ninety-eight degrees is the natural temperature of the blood—that serious results do not follow exposure to a degree of heat almost sufficient to cook the blood in the veins.—*Virginia City (Nev.) Enterprise.*

